# Fully human antibody V_H_ domains to generate mono and bispecific CAR to target solid tumors

**DOI:** 10.1136/jitc-2020-002173

**Published:** 2021-04-01

**Authors:** Guanmeng Wang, Xin Zhou, Giovanni Fucà, Elena Dukhovlinova, Peishun Shou, Hongxia Li, Colette Johnston, Brian Mcguinness, Gianpietro Dotti, Hongwei Du

**Affiliations:** 1 Lineberger Comprehensive Cancer Center, University of North Carolina at Chapel Hill, Chapel Hill, North Carolina, USA; 2 Crescendo Biologics Ltd, Cambridge, UK; 3 Department of Microbiology and Immunology, University of North Carolina at Chapel Hill, Chapel Hill, North Carolina, USA

**Keywords:** immunotherapy, tumor escape, MSLN, PSMA, Humabody, CAR-T

## Abstract

**Background:**

Chimeric antigen receptor (CAR) T cells are effective in B-cell malignancies. However, heterogeneous antigen expression and antigen loss remain important limitations of targeted immunotherapy in solid tumors. Therefore, targeting multiple tumor-associated antigens simultaneously is expected to improve the outcome of CAR-T cell therapies. Due to the instability of single-chain variable fragments, it remains challenging to develop the simultaneous targeting of multiple antigens using traditional single-chain fragment variable (scFv)-based CARs.

**Methods:**

We used Humabody V_H_ domains derived from a transgenic mouse to obtain fully human prostate-specific membrane antigen (PSMA) V_H_ and mesothelin (MSLN) V_H_ sequences and redirect T cell with V_H_ based-CAR. The antitumor activity and mode of action of PSMA V_H_ and MSLN V_H_ were evaluated in vitro and in vivo compared with the traditional scFv-based CARs.

**Results:**

Human V_H_ domain-based CAR targeting PSMA and MSLN are stable and functional both in vitro and in vivo. V_H_ modules in the bispecific format are capable of binding their specific target with similar affinity as their monovalent counterparts. Bispecific CARs generated by joining two human antibody V_H_ domains can prevent tumor escape in tumor with heterogeneous antigen expression.

**Conclusions:**

Fully human antibody V_H_ domains can be used to generate functional CAR molecules, and redirected T cells elicit antitumoral responses in solid tumors at least as well as conventional scFv-based CARs. In addition, V_H_ domains can be used to generate bispecific CAR-T cells to simultaneously target two different antigens expressed by tumor cells, and therefore, achieve better tumor control in solid tumors.

## Introduction

Chimeric antigen receptors (CARs) typically consist of an extracellular antigen-binding domain in the form of a single-chain fragment variable (scFv), a transmembrane domain and signaling molecules such as costimulatory endodomains and CD3ζ chain.^1–3^ Expression of CARs in T cells enables specific targeting of surface antigens in a Major Histocompatibility Complex-independent manner and associated T cell activation.^4 5^ While classical CARs use scFvs as the antigen-binding moiety, other ligands fused with signaling molecules of the T-cell receptor complex can also trigger phosphorylation events in T cells.^6^ For example, engineering of natural receptors such as NKG2D and CD27 fused with CD3ζ have been shown to redirect T cell specificity.^7 8^ Ligands to receptors such as interleukin (IL)-13Rα2 have also been engineered to redirect T cell specificity towards glioblastoma.^9^ More recently, synthetic antigen binding moieties as exemplified by a ‘monobody’ based on the type III domain of fibronectin have also been shown to serve as a robust platform to generate CAR molecules.^10^ Therefore, using alternative binding moieties to replace scFvs to generate CAR remains a critical area because scFvs are frequently unstable and showing intrinsic tendency to self-aggregation, which may lead to tonic signaling and loss of function of CAR-T cells in vivo.^11 12^

A scFv molecule is composed of paired antibody (Ab) light chain and heavy chain variable domains (V_L_ and V_H_) that are fused into a single polypeptide chain via a short flexible linker.^11 13 14^ Heavy-chain-only Abs without light chains have been reported in camelids and cartilaginous fish,^15–17^ and shown to exhibit strong and specific antigen binding.^13^ Single domains targeting BCMA (B-cell maturation antigen) have been developed to generate BCMA-specific CAR-T cells for the treatment of multiple myeloma.^18^ Whether human-derived V_H_-only domains can be used as a CAR to target antigens expressed in solid tumors is unknown.

Treatment failure and/or disease recurrence after CAR-T cell therapy can be caused by epitope or antigen loss.^10^ In particular, the inherently heterogeneous expression pattern of antigens in solid tumors can easily cause tumor escape after targeted immunotherapy.^10 19 20^ Therefore, targeting multiple tumor-associated antigens (TAAs) is generally expected to improve the outcome of CAR-T cell therapy in solid tumor.^10 19^ However, including multiple scFvs within a CAR causes protein instability and decreases binding specificity and affinity. V_H_ domain-only format of CARs provide an ideal solution for multiple antigen targeting because V_H_ domains have smaller size and may easily fold correct 3D structure compared to scFv molecules.

Here, we explored the use of Humabody V_H_ domains derived from a transgenic mouse to develop CARs that target prostate-specific membrane antigen (PSMA)^21^ ^22^ and mesothelin (MSLN).^23^ We found that Humabody-based CARs exhibited comparable or superior antitumor activity compared with traditional scFv CARs. Moreover, we demonstrated that Humabodies were suitable for constructing bispecific CAR-T cells, which can significantly better control tumors with heterogeneous antigen expression.

## Materials and methods

### Generation of V_H_ domains

Crescendo Mouse^17^ was immunized with PSMA and MSLN recombinant proteins. Spleens and lymph nodes were harvested, cloned into a phagemid vector and selected by phage display. Outputs were screened for specific target binding and further characterized.

### CAR construction

The following antigen-binding moieties were used: scFv derived from the J591 Ab specific for PSMA; human V_H_ domain specific for PSMA (PSMA-V_H_); scFv derived from a MSLN-specific Ab Amatuximab; human V_H_ domain specific for MSLN (MSLN-V_H_). All ligands were assembled with the CD8α hinge and transmembrane domain, the CD28 costimulatory domain and CD3ζ intracellular signaling domain and cloned into the SFG retroviral vector.^24^ A FLAG-tag was incorporated after the antigen ligand to detect the expression of CARs by an anti-FLAG Ab. Dual specific (PSMA and MSLN) CARs were also generated by linking the two V_H_ domains. The corresponding CARs were called J591, PSMA-V_H_, MSLN scFv, MSLN-V_H_ and PSMA-V_H_/MSLN-V_H_. Retroviral supernatants were produced by transfection of 293 T cells with the retroviral vectors, the RD114 envelope from RDF plasmid and the MoMLV gag-pol from PegPam3-e plasmid. Supernatants were collected 48 hours and 72 hours after the transfection and filtered with 0.45 µm filter.^24^

### Cell lines

Tumor cell lines PC-3, C4-2 (prostate cancer) and Aspc-1 (pancreatic cancer) were purchased from ATCC (American Type Culture Collection). All tumor cell lines were cultured with RPMI-1640 (Gibco) supplemented with 10% Fetal bovine serum (Sigma), 2 mM GlutaMax (Gibco) and penicillin (100 units/mL) and streptomycin (100 µg/mL; Gibco). All cells were cultured at 37°C with 5% CO_2_. PC-3 cell line was transduced with retroviral vectors encoding PSMA or MSLN to make PC-3-PSMA and PC-3-MSLN. PC-3-PSMA, PC-3-MSLN and Aspc-1 were transduced with retroviral vectors encoding Firefly-Luciferase-eGFP (FFluc-eGFP) gene.

### CAR-T cell generation

Buffy coats from healthy donors (Gulf Coast Regional Blood Center) were processed with Lymphoprep density separation (Fresenius Kabi Norge) to isolate peripheral blood mononuclear cells, which were then activated on plates coated with 1 µg/mL CD3 (Miltenyi Biotec) and 1 µg/mL CD28 (BD Biosciences) monoclonal Abs (mAbs). Two days later, activated T cells were transduced with retroviral supernatants on 24-well plates coated with retronectin (Takara Bio). T cells were collected 3 days after transduction and expanded in 40% RPMI-1640(Gibco) and 40% Click’s medium (Irvine Scientific), 10% HyClone FBS (GE healthcare), 2 mM GlutaMAX(Gibco), 100 unit/mL of Penicillin and 100 mg/mL of streptomycin (Gibco) with 10 ng/mL IL-7 (PeproTech) and 5 ng/mL IL-15 (PeproTech). T cells were collected for functional assays 12–14 days after activation.^25 26^

### Flow cytometry

mAbs for human CD3 (APC-H7; SK7; 560176), CD4 (BV711; SK3; 563028), CD8 (APC; SK1; 340584), CD45RA (PE; HI100; 555489), CD45RO (BV786; UCHL1; 564290), CD69 (FITC; L78; 347823), CCR7 (FITC; 150503; FAB197F-100), PD-1(PE-Cy7; EH12.1;561272), Lag3(PE;T47-530;565616), FLAG (APC; L5; 637308), Granzyme-B (PE;GB11;561142) from BD biosciences and BioLegend were used. Samples were acquired with BD FACSCanto II or BD LSRFortessa. A minimum of 10 000 events were acquired for each sample and were analyzed using FlowJo 10 (FlowJo).

### Western blot

CAR-T cells were incubated with 2 µg anti-FLAG Ab in 100 µL PBS for 20 mins on ice and then with 2 µg goat antimouse secondary Ab for another 20 mins on ice. Cells were then incubated in the 37°C water bath for the selected time points and then lysed with 2 x Laemelli buffer for 10 mins. Cell lysates were then separated in 4% to 15% 10 well SDS-PAGE gels and transferred to polyvinylidene difluoride membranes at 75V for 120 mins (Bio-Rad). Blots were examined for human CD3ζ (Santa Cruz Biotechnology), p-Y142 CD3ζ (Abcam), pan-ERK (BD Biosciences), and pan-Akt, p-S473 Akt, and p-T202/Y204 MAPK (Cell Signaling Technology) with 1:1000 dilution in 5% TBS-Tween milk. Membranes were incubated with HRP-conjugated secondary goat anti-mouse or goat anti-rabbit IgG (Santa Cruz) at a dilution of 1:3000 and imaged with the ECL substrate kit (Thermofisher) on the ChemiDoc MP System (Bio-Rad) according to the manufacturer’s instructions.^26^

### Proliferation assay

T cells were labeled with 1.5 mM carboxyfluorescein diacetate succinimidyl ester (CFSE; Invitrogen) and plated with tumor cells at an effector to target (E:T) ratio of 1:1. CFSE signal dilution from gated T cells on day 5 was measured using flow cytometry.^26^

### In vitro cytotoxicity assay

Tumor cells were seeded in 24-well plates at a concentration of 2.5×10^5^ cells/well overnight. CAR-T cells were added to the plate at an E:T of 1:5 without exogenous cytokines. Cocultures were analyzed 5–7 days following coculture to measure residual tumor cells and T cells by flow cytometry. Dead cells were recognized by Zombie Aqua Dye (Biolegend) staining while CAR-T cells were identified by CD3 staining and tumor cells by GFP.^26^ CD69, PD-1 and Lag3 expression was measured by flow cytometry from day 0 to day 5 each day after coculture of CAR-T cells with tumor cells. For the granzyme-B staining, Golgi protein inhibitor (BD Biosciences) was added on day 1 of coculture for 6 hours. Cocultures were then first stained with Zombie Aqua Dye (Biolegend) and CD3 mAb, followed by fixation/permeabilization solution (BD Biosciences). Intracellular staining of granzyme-B was then conducted.

### Cytokine analysis

CAR-T cells (1×10^5^ cells) were cocultured with 2.5×10^5^ tumor cells in 24-well plates without exogenous cytokines. Supernatant was collected after 24 hours, and cytokines (interferon-γ (IFN-γ) and IL-2) were measured by using ELISA kits (R&D, Research And Development system) in duplicates following manufacturer’s instructions.^26^

### Expression and purification of recombinant proteins

A panel of recombinant proteins was produced, comprizing bispecific (2V_H_) proteins that bind both PSMA and MSLN, monospecific V_H_ protein binding PSMA, monospecific V_H_ protein binding MSLN and a control scFv protein based on Amatuximab. Bispecific protein was made in two formats, one with a short flexible linker (G4S)_3_, aother one with a long flexible linker (G4S)_6_. Bispecific proteins were expressed in mammalian cells and purified by protein A binding. Monospecific proteins were His tagged at the C terminus, expressed in *Escherichia coli* and purified by His trap and size exclusion chromatography.

### Binding and kinetic analysis

Binding analyses were performed at 25°C using BIAcore 8K system. The instrument was run on 1 x HBS-EP^+^ (BR100669) buffer and the data were analyzed using Biacore Insight Evaluation software. Recombinant human MSLN was diluted to 2 ug/mL in 10 mM sodium acetate buffer pH4.0 and immobilized on a CM5 sensor chip (contact time 120 s) using amine-coupling kit with accordance to the manufacturer’s instructions. Humabody V_H_ samples were tested for binding at 5 concentrations 3.7 nM, 11.1 nM, 33.3 nM, 100 nM and 300 nM using multicycle kinetics method. Each sample was injected for 100 s at the flow rate 35 µL/min and dissociated for 100 s. The antigen surface was regenerated by 20 s injection of 10 mM glycine pH 2.0. Recombinant human PSMA antigen with a human Fc tag was captured on a Protein G sensor. Humabody V_H_ samples were tested in Single-cycle kinetics mode at increasing concentrations of 2.22 nM, 6.67 nM, 20 nM and 60 nM with 90 s association and 600 s dissociation time at the flow rate of 30 µL/min. Buffer injections were made to allow for double-reference subtraction. The sensor surface was regenerated with 10 mM glycine pH1.5 (GE Healthcare BR100354). To detect dual binding to MSLN and PSMA, human PSMA antigen surface was captured as above. Bispecific PSMA-MSLN Humabody constructs were captured on the PSMA surface by injecting 100 nM of each sample for 100 s at 35 µL/min flow rate. The capture was immediately followed by an injection of 300 nM recombinant human MSLN with 100 s contact time and 100 s dissociation. A PSMA-specific Humabody construct without a MSLN-binding arm was used as a control.

### Xenograft murine models

NSG (NOD scid gamma mouse) mice (6–8 weeks old) were injected intravenously through tail vein with either PC-3-PSMA-FFluc-eGFP, or PC-3-PSMA-FFluc-eGFP and PC-3-MSLN-FFluc-eGFP mixed at 1 to 1 ratio, or Aspc-1-FFluc-eGFP tumor cells of 1×10^6^ cells per mice. Fourteen days later, CAR-T cells were injected intravenously through tail vein. For the high dose treatment, 4×10^6^ CAR-T cells per mice were injected, while for the low dose treatment, 1×10^6^ CAR-T cells per mice were injected. In the rechallenge experiments, mice were infused 1×10^6^ tumor cells per mice on clearance of the previous tumor. Tumor growth was monitored by bioluminescence using IVIS (In Vivo Imaging Systems)-Kinetics Optical in vivo imaging system (PerkinElmer) (PSMA-V_H_ and MSLN-V_H_ part) or AMI(AMI Medical Imaging) Optical in vivo imaging system (Spectral instruments imaging) (PSMA-V_H_/MSLN-V_H_ part).

### Statistics

All data was calculated and represented as mean with SD. One-way analysis of variance (ANOVA) or two-way ANOVA analyses were performed to compare multiple groups. Two-tailed t-test was used to compare two groups. P value of less than 0.05 was significant. All calculations and figures were achieved by GraphPad Prism V.7 (La Jolla, California, USA).

## Results

### Human V_H_ domain-based CAR targeting PSMA is expressed and signals in T cells

**Figure 1:**
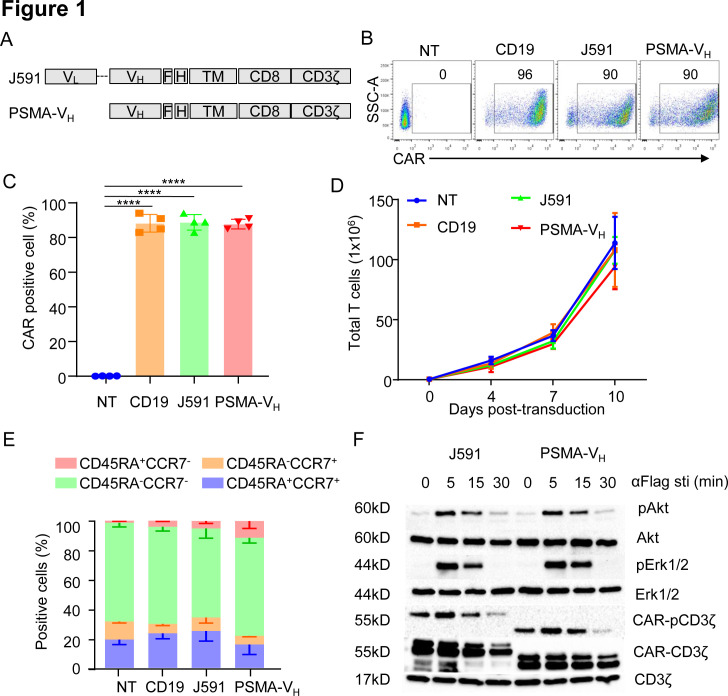
Human antibody V_H_ domain-based CAR targeting PSMA is expressed and signals in T cells. (A) Schematic diagram of J591 and PSMA-V_H_ constructs. (B, C) Representative flow cytometry plots (B) and summary (C) illustrating J591 and PSMA-V_H_ expression in T cells. The CD19-specific CAR (CD19) and non-transduced T cells (NT) were used as positive and negative controls, respectively. ****P<0.0001, one-way ANOVA. (D) In vitro expansion of CD19, J591, PSMA-V_H_ and NT T cells; error bars represent SD, (n=4). P≥0.05 by one-way ANOVA. (E) T cell subset composition based on CD45RA and CCR7 expression in CD19, J591, PSMA-V_H_ and nt T cells at day 14 of culture; error bars represent SD, (n=4). P≥0.05 by one-way ANOVA. (F) Western blots detecting phosphorylation of CAR-CD3ζ, Akt and ERK in J591 and PSMA-V_H_ T cells activated via CAR cross-linking with an anti-FLAG ab followed by incubation with a secondary ab to induce the aggregation of car molecules. Total CAR.CD3ζ and endogenous CD3ζ were used as loading controls. Data are representative of two experiments. ANOVA, analysis of variance; CAR, chimeric antigen receptor; MSLN, mesothelin; PSMA, prostate-specific membrane antigen.

We constructed the PSMA-specific CARs using the scFv from the J591 mAb (J591) and the PSMA binding human V_H_ domain (PSMA-V_H_) joined to the CD8α stalk, CD28 costimulatory domain and CD3ζ intracellular domain. A flag-based tag was incorporated into the cassettes to detect CAR expression by flow cytometry (figure 1A). Activated T cells were successfully transduced and expressed the CARs equally (figure 1B, C). The CD19-specific CAR (CD19) and non-transduced (NT) T cells were used as controls. On transduction, J591-T cells and PSMA-V_H_-T cells showed similar expansion in vitro when exposed to IL-15 and IL-7 cytokines, which was similar to CD19-T cells and NT-T cells (figure 1D). Furthermore, no differences were observed in T cell composition as assessed by flow cytometry at day 12–14 of culture (figure 1E). We examined proximal signaling of CAR-T cells before and after CAR cross-linking mediated by an anti-Flag Ab. Phosphorylation of the CAR-associated CD3ζ as well as phosphorylation of Akt and ERK were equal in J591-T cells and PSMA-V_H_-T cells (figure 1F). Therefore, a V_H_ domain-based CAR is expressed and signals in T cells on cross-linking as observed for scFv-based CAR-T cells.

### PSMA-specific V_H_ domain-based CAR-T cells are functional in vitro and in vivo

**Figure 2:**
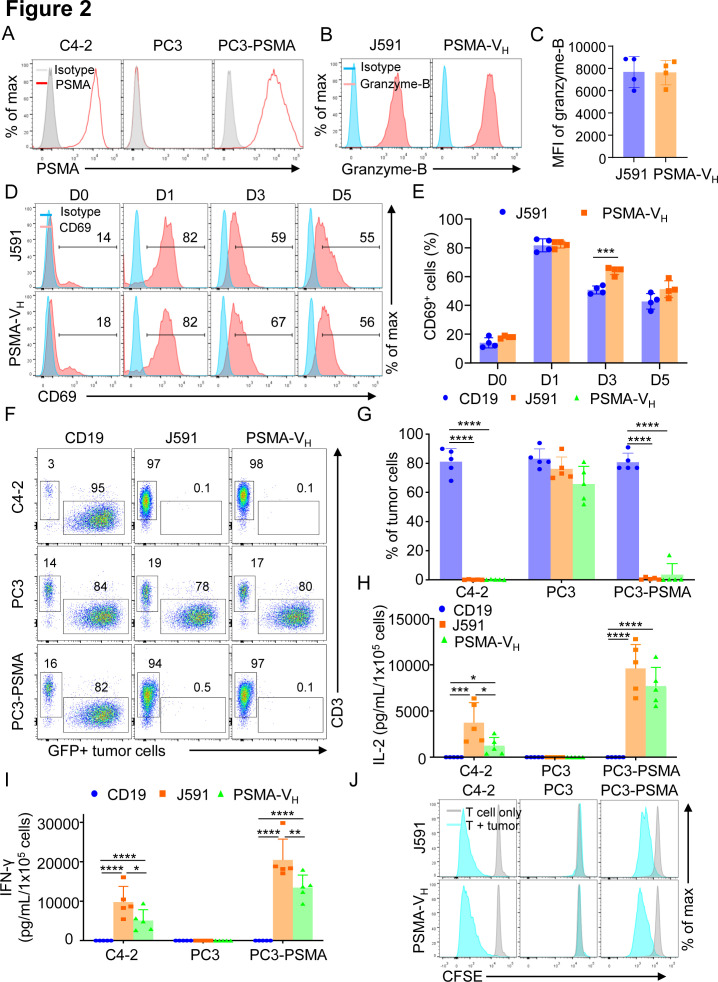
T cells expressing the human antibody V_H_ domain-based CAR targeting PSMA are functional in vitro. (A) Representative flow cytometry plots showing the expression of PSMA in C4-2, PC3 and PC3 cells engineered with a retroviral vector to express PSMA. (B, C) Rpresentative flow cytometry plots (B) and summary (C) illustrating Granzyme-B expression of T cells expressing either J591 or PSMA-V_H_ cocultured overnight with a tumor cell line expressing PSMA (PC3-PSMA-eGFP) at E:T ratio of 1:2; error bars represent SD, (n=4). P≥0.05 by t-test. (D, E) Representative flow cytometry plots (D) and summary (E) illustrating the kinetics of CD69 expression of T cells expressing either J591 or PSMA-V_H_ and cocultured overnight with a tumor cell line expressing PSMA (PC3-PSMA-eGFP) at E:T ratio of 1:2. Data are representative of 4 experiments. ***P<0.001 two-way ANOVA. (F) Representative flow cytometry plots showing coculture of CD19, J591 and PSMA-V_H_ T cells with C4-2-eGFP, PC3-eGFP and PC3-PSMA-eGFP. T cells were cocultured with tumor cells at an E:T ratio of 1:5 for 6 days. At day 6, all cells were collected and analyzed by flow cytometry to quantify tumor cells (GFP) and T cells (CD3), respectively. (G) Summary of coculture of CD19, J591 and PSMA-V_H_ T cells with tumor cells in (F); error bars represent SD, (n=4). ****P<0.0001, two-way ANOVA. (H, I) IFN-γ (H) and IL-2 (I) were detected by ELISA in the coculture supernatant of cocultures of CD19, J591 and PSMA-V_H_ T cells with tumor cells illustrated in (F); error bars represent SD, (n=5). *P<0.05, **p<0.01, ***p<0.001, ****p<0.0001, two-way ANOVA. (J) Representative flow cytometry plots showing the proliferation of J591 and PSMA-V_H_ T cells in response to tumor cells as assed by CFSE dilution. Data are representative of four experiments. ANOVA, analysis of variance; CAR, chimeric antigen receptor; E:T, effector to target ratio; IFN-γ, interferon-γ; IL-2, interleukin 2; MSLN, mesothelin; PSMA, prostate-specific membrane antigen.

**Figure 3:**
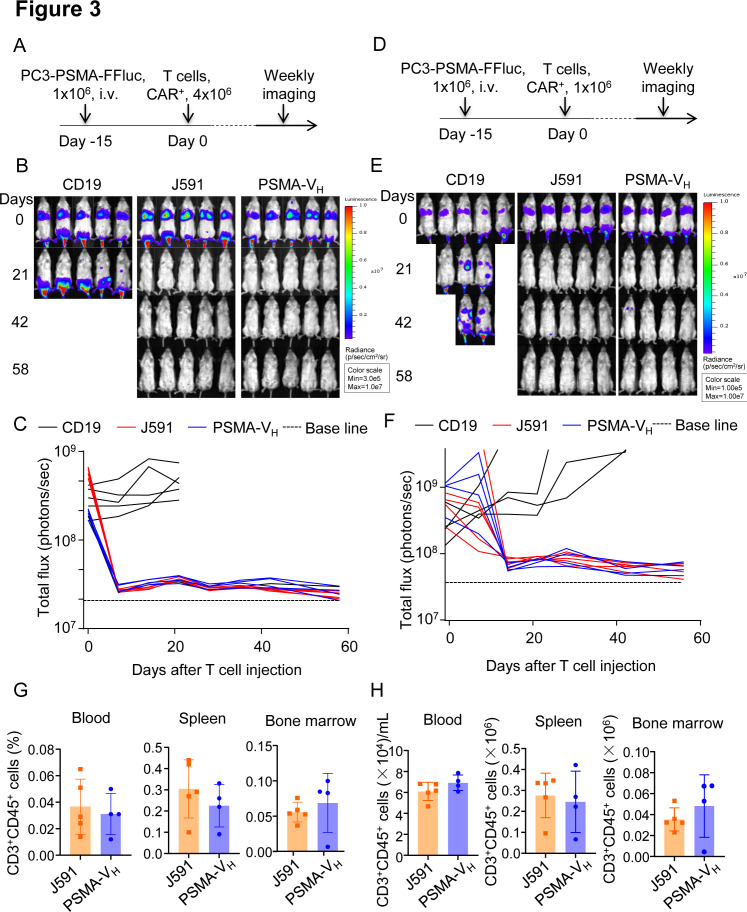
T cells expressing the human antibody V_H_ domain-based CAR targeting PSMA are functional in vivo. (A) Schematic of the metastatic prostate cancer model using PC3-PSMA-FFluc-eGFP tumor cells in NSG mice and treatment with CD19, J591 and PSMA-V_H_ T cells (n=5 mice per group). (B) Representative images of tumor bioluminescence (BLI) at selected time points post T cell injections. (C) Kinetics of tumor BLI post T cell injections. (D) Schematic of the metastatic prostate cancer model using PC3-PSMA-FFluc-eGFP tumor cells in NSG mice and treatment with low dose of CD19, J591 and PSMA-V_H_ T cells (n ≥4 mice per group). (E) Representative images of tumor BLI at selected time points post-T cell injections for low dose of T cells. (F) Kinetics of tumor BLI post T cell injections low dose of T cells. (G, H) Percentage of T cells in the gate of live cells (G) and total cell numbers (H) in blood, spleen and bone marrow from PC3-PSMA-bearing mice treated with low doses of CAR-T cells. Mice were euthanized at day 58 after CAR-T cells infusion and T cells were identified as CD45^+^CD3^+^ cells by flow cytometry. J594 group (n=5), PSMA-V_H_ group (n=4). P≥0.05 by t-test. CAR, chimeric antigen receptor; BLI, bioluminescence; PSMA, prostate-specific membrane antigen.

To compare the antitumor effect of PSMA-V_H_-T cells and J591-T cells in vitro, we engineered the PC3 cells to express PSMA antigen (figure 2A), and we observed that J591-T cells and PSMA-V_H_-T cells showed comparable granzyme-B expression when cultured with or without tumor cells (figure 2B, C and online supplemental figure S1A). Similarly, both PSMA-V_H_-T cells and J591-T cells showed equal upregulation and subsequent down regulation of CD69 as a marker of T cell activation (figure 2D). Similar upregulation on antigen stimulation and down regulation after antigen removal were observed for PD-1 and Lag-3 (online supplemental figure S1B-E). We then cocultured PSMA-V_H_-T cells and J591-T cells in vitro with tumor cells (either PSMA-positive or PSMA-negative), and measured the remaining tumor cells after 5 days of coculture. CD19-T cells did not eliminate tumor cells, while PSMA-V_H_-T cells specifically eliminated PSMA-positive target cells (C4-2 and PC3-PSMA) to the same extent as conventional J591-T cells, and did not demonstrate off-target effect on PSMA-negative cells (PC3) (figure 2F, G). We also measured the secretion of IFNγ and IL-2 after 24 hours of coculture with tumor cells. When the PSMA-V_H_-T and J591-T cells target PSMA-positive C4-2 and PC3-PSMA cells, both of them secreted high amount of IFNγ and IL-2 compared with control CD19-CAR-T cells (figure 2H, I). Furthermore, PSMA-V_H_-T cells and J591-T cells proliferated similarly on encounter with tumor cells as shown by CFSE dilution (figure 2J). To investigate the antitumor effects of Humabody V_H_ CAR-T cells in vivo, NSG mice engrafted with PSMA-positive tumor cells labeled with Firefly luciferase were treated with a high doses (4×10^6^ cells/mouse) of CAR-T cells (figure 3A). CAR-T cell treatment showed tumor control as measured by tumor bioluminescence without differences in mice treated with PSMA-V_H_-T cells or J591-T cells (figure 3B, C). To further assess differences between PSMA-V_H_-T cells and J591-T cells, we used low doses of T cells (1×10^6^ cells/mouse) in tumor-bearing mice (figure 3D). We observed that PSMA-V_H_-T cells still eliminated tumor cells in vivo as J591-T cells (figure 3E, F). In addition, we also observed similar V_H_ CAR-T cell persistence in the peripheral blood, spleen and bone marrow compared with traditional scFv-based CAR-T cells at day 58 at the time of euthanasia (figure 3G, H). Therefore, Humabody V_H_ CAR-T cells demonstrated comparable antitumor effects to scFv-based CAR-T cells in vitro and in vivo.

10.1136/jitc-2020-002173.supp1 Supplementary data

10.1136/jitc-2020-002173.supp6 Supplementary data

### MSLN-specific V_H_ domain-based CAR-T cells demonstrate antitumor activity

**Figure 4:**
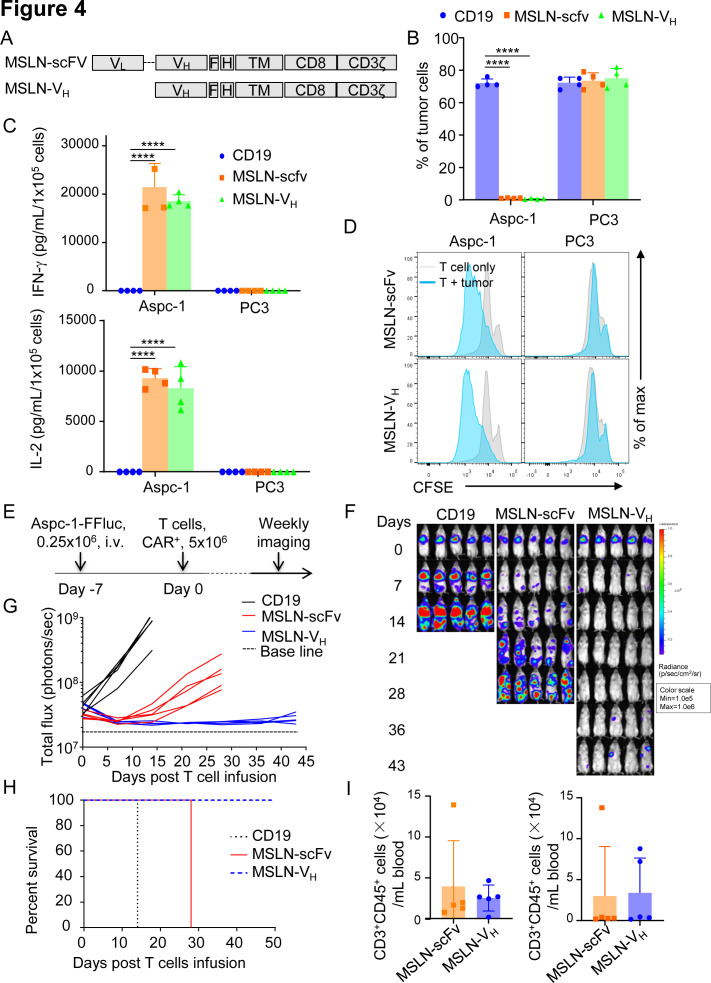
T cells expressing the human antibody V_H_ domain-based CAR targeting MSLN demonstrate antitumor activity. (A) Schematic diagram of MSLN-scFv and MSLN-heavy-chain-only (MSLN-V_H_) CAR constructs. (B) Summary of coculture of CD19, MSLN.scFv and MSLN-V_H_ T cells with Aspc-1-eGFP (MSLN^+^) and PC3-eGFP (MSLN^-^) tumor cell lines. T cells were cocultured with tumor cells at an E:T ratio of 1:5 for 6 days. At day 6, all cells were collected and analyzed by flow cytometry to quantify tumor cells and T cells, respectively. Error bars represent SD, (n=4). ****P<0.0001, two-way ANOVA. (C) IFN-γ (upper panel) and IL-2 (lower panel) detected in the supernatants of the cocultures illustrated in (B) as measured by ELISA; error bars represent SD, (n=4). ****P<0.0001, two-way ANOVA. (D) Representative flow cytometry plots showing the proliferation of MSLN.scFv and MSLN-V_H_ T cells in response to tumor cells as assessed by CFSE dilution. Data are representative of three experiments. (E) Schematic of the metastatic pancreatic cancer model using Aspc-1-FFluc-eGFP tumor cells in NSG mice. (F, G) Representative tumor BLI (F) and BLI kinetics (G) of Aspc-1-FFluc-eGFP tumor growth at the representative time points post T cell injections. (n=5 mice per group). (H) Kaplan-Meier survival curve of mice in (E) (n=5 mice per group). Data are representative of two experiments. (I) Frequency of human CD45^+^CD3^+^cells in blood at 22 days (left) post-T-cell infusion and at euthanasia (right) of MSLN-scFv and MSLN-V_H_ T cells, respectively. Data are shown as individual values and the mean (n = 5 mice per group). P≥0.05 by t-test. ANOVA, analysis of variance; BLI, bioluminescence; CAR, chimeric antigen receptor; CFSE, carboxyfluorescein diacetate succinimidyl ester; E:T, effector to target ratio; IFN-γ, interferon-γ; IL-2, interleukin 2; i.v, intravenous; MSLN, mesothelin; scFv, single-chain fragment variable.

To further assess the reproducibility of V_H_ domain-based CARs, we tested a MSLN-specific Humabody V_H_. We constructed the conventional MSLN scFv CAR (MSLN-scFv) and V_H_ domain CAR (MSLN-V_H_) using the same backbone developed for PSMA-specific CARs (figure 4A). MSLN-scFv and MSLN-V_H_ were equally expressed in T cells (online supplemental figure S2A, B). In a similar fashion, we examined the antitumor activity of MSLN-scFv-T cells and MSLN-V_H_-T cells in vitro through coculture experiments with tumor cells, cytokine release assay, proliferation assay. MSLN-scFv-T cells and MSLN-V_H_-T cells selectively eliminated Aspc-1 tumor cells that express MSLN, while spared PC3 cells that do not express MSLN (figure 4B and online supplemental figure S3). They released similar amount of IFNγ and IL-2 (figure 4C) and proliferated on encounter with tumor cells (figure 4D). In the xenotransplant model in NSG mice engrafted with Aspc-1 cells labeled with Firefly luciferase (figure 4E), MSLN-V_H_-T cells showed even more profound antitumor effects as compared with mice treated with MSLN-scFv CAR-T cells (figure 4F, G), which translated in prolonged survival of the mice (figure 4H). However, we observed similar T cells expansion/persistence between MSLN-V_H_ and MSLN-ScFv (figure 4I). Thus V_H_ domain-based CARs can reproducibility redirect antitumor activity of engineered T cells.

10.1136/jitc-2020-002173.supp2 Supplementary data

10.1136/jitc-2020-002173.supp3 Supplementary data

### In vitro analysis of monovalent and bivalent V_H_ domain recombinant proteins

**Figure 5:**
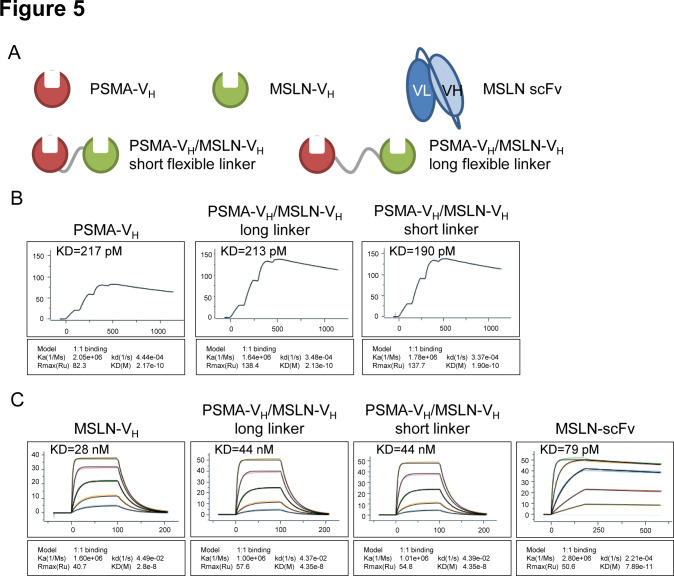
In vitro analysis of monospecific and bispecific Humabody V_H_ binding. (A) Schematic representation of monospecific (single V_H_) or bispecific (double V_H_) proteins. (B) Single cycle BIAcore kinetic analysis of PSMA binding. (C) BIAcore kinetic analysis of MSLN binding, threefold dilution series starting at 300 nM, except the control scFv protein which started at 33.3 nM. Data are representative of two experiments. MSLN, mesothelin; PSMA, prostate-specific membrane antigen; scFv, single-chain fragment variable.

To test whether the V_H_ domains are suitable to construct bispecific CARs, two V_H_ domains in tandem recombinant proteins linking PSMA-specific and MSLN-specific V_H_ were generated (figure 5A). To test whether the linkers had any effect on the target binding affinity, two different linkers were used: the (G4S)_3_ linker (‘short flexible linker’) and a longer linker (G4S)_6_ with 6 copies of the (G4S) repeat (‘long flexible linker’). Monomer V_H_ proteins and a MSLN binding scFv were made as controls (figure 5A). Analysis of binding to PSMA recombinant protein by surface plasmon resonance (SPR) Biacore assay showed that the affinity of the PSMA-V_H_ remained the same when the PSMA-V_H_ was formatted with the MSLN-V_H_ domain using either flexible linkers (figure 5B). Similarly, analysis of binding to MSLN recombinant protein by SPR Biacore assay showed that the affinity of the MSLN-V_H_ domain was not altered when the PSMA-V_H_ was formatted with the MSLN-V_H_ using either flexible linkers (figure 5C). In summary, these data show that V_H_ modules in bispecific format are capable of binding their specific target with the same affinity as their monovalent counterparts.

### Bispecific V_H_ domain-based CAR-T cells demonstrate dual specificity

**Figure 6:**
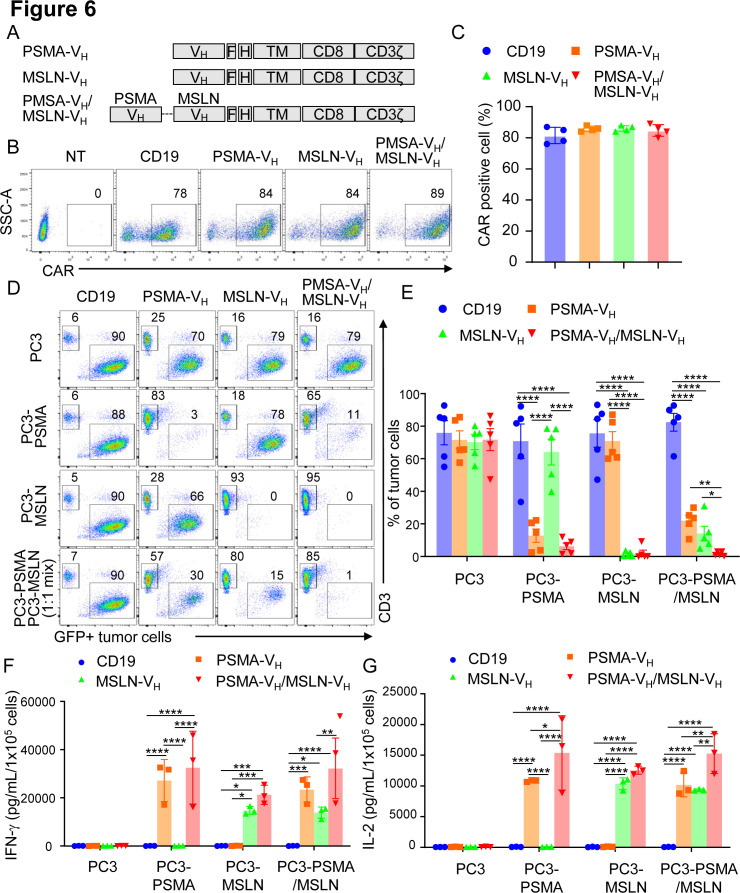
T cells expressing two human antibody V_H_ domain-based CARs demonstrate dual specificity in vitro. (A) Schematic diagram of PSMA-V_H_, MSLN-V_H_, and PSMA/MSLN-V_H_ CAR constructs. (B, C) Representative flow cytometry plots (B) and summary (C) illustrating CAR expression in T cells. The CD19-specific CAR (CD19) was used as negative controls. P≥0.05 by one-way ANOVA. (D) Representative flow cytometry plots showing PC3-PSMA-eGFP (PSMA target), PC3-MSLN-eGFP (MSLN target) and mixture of PC3-PSMA-eGFP and PC3-MSLN-eGFP (1:1 ratio) cotultured with CD19.CAR, PSMA-V_H_.CAR, MSLN-V_H_.CAR and PSMA/MSLN-V_H_.CAR T cells at the E:T ratio of 1:5 for 6 days. Tumor cells and T cells were quantified at day six by flow cytometry. (E) Summary of coculture experiments illustrated in (D); error bars represent SD, (n=5). *P<0.05, **p<0.01, ****p<0.0001, two-way ANOVA. (F, G) IFN-γ (F) and IL-2 (G) detected in the coculture supernatant of the coculture experiments described in (D) as measured by ELISA; error bars represent SD, (n=3) *p<0.05, **p<0.01, ***p<0.001, ****p<0.0001, two-way ANOVA. ANOVA, analysis of variance; CAR, chimeric antigen receptor; E:T, effector to target ratio; IFN-γ, interferon-γ; IL-2, interleukin; MSLN, mesothelin; NT, non-transduced; PSMA, prostate-specific membrane antigen.

**Figure 7:**
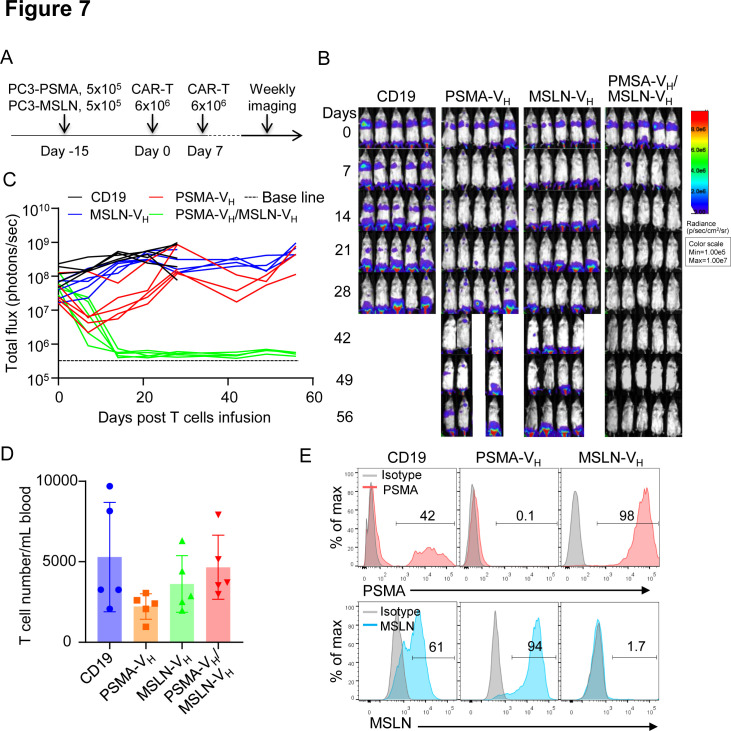
T cells expressing two human antibody V_H_ domain-based CARs demonstrate dual specificity in vivo. (A) Schematic of the xenograft mouse model in which NSG mice were systemically engrafted with mixed FFluc-eGFP labeled PC3-PSMA (5×10^5^ cells) and PC3-MSLN (5×10^5^ cells) cells at 1:1 ratio, and treated with two doses of CAR-T cells at day 0 and day 7, respectively (6×10^6^ cells each dose, n=5 mice per group). (B, C) Representative tumor BLI images (B) and BLI kinetics (C) at selected time points post T cell injections. (D) Number of human CD45^+^CD3^+^ cells in the peripheral blood collected at day 21 post second T-cell infusion in mice treated as described in (A). Data are shown as individual values and the mean (n=5 mice per group) and are representative of two experiments, p≥0.05 by one-way ANOVA. (E) Representative antigen expression pattern in the tumor cells isolated from the mice with relapsed tumor in mice treated as described in (A). ANOVA, analysis of variance; BLI, bioluminescence; CAR, chimeric antigen receptor; MSLN, mesothelin; PSMA, prostate-specific membrane antigen.

We constructed a bispecific V_H_ domain CAR to facilitate CAR-T cells to specifically recognize two antigens simultaneously. We used the MSLN-V_H_ and PSMA-V_H_ domains fused with the short (G4S)_3_ linker to generate the bispecific PSMA-V_H_/MSLN-V_H_ CAR (figure 6A). The PSMA-V_H_/MSLN-V_H_ CAR was expressed in T cells (figure 6B, C). PSMA-V_H_-T cells, MSLN-V_H_-T cells and PSMA-V_H_/MSLN-V_H_-T cells were cocultured with tumor the cell line Aspc-1, which express MSLN, and the PC3-PSMA cell line. We observed the PSMA-V_H_/MSLN-V_H_-T can eliminate both tumor cell lines compared with single CAR-T cells, which only eliminate tumor cells expressing the targeted antigen (online supplemental figure S4A, B). In addition, we also observed the expected cytokine release profile (online supplemental figure S4C, D). Next, we confirmed that PSMA-V_H_/MSLN-V_H_-T cells displayed specific cytotoxicity toward the same cell line PC3 expressing either MSLN or PSMA similar to MSLN-V_H_-T cells and PSMA-V_H_-T cells without off-target effect (figure 6D, E). Importantly, when PC3-PSMA and PC3-MSLN were plated as 1:1 ratio mixture in coculture experiments, only PSMA-V_H_/MSLN-V_H_-T cells fully eliminated the tumor cells, although PSMA-V_H_-T cells and MSLN-V_H_-T cells showed some bystander killing effect as previously observed^27^ (figure 6D, E). The in vitro antitumor effect was corroborated by release of IFN-γ and IL-2 (figure 6F, G). To evaluate if bispecific V_H_ domain CAR-T cells can eradicate tumors with mixed antigen expression in vivo, we established a metastatic xenograft mouse model by infusing PC3-PSMA cells and PC3-MSLN cells at 1:1 ratio into NSG mice by intravenous injection. Mice were then treated with CAR19-T, PSMA-V_H_-T, MSLN-V_H_-T and PSMA-V_H_/MSLN-V_H_-T cells (figure 7A). Dual targeting PSMA-V_H_/MSLN-V_H_-T cells controlled the tumor growth more effectively than either single targeting PSMA-V_H_-T or MSLN-V_H_-T cells (figure 7B, C). CAR-T cells were detectable in the peripheral blood of these mice up to 4 weeks after infusion (figure 7D). We also observed that T cells expressing bispecific CAR showed similar phenotypic profile as single CAR targeting T cells for exhaustion and memory markers (online supplemental figure S5A-C). Analyses of antigen expression in tumor cells in vivo showed that tumor cells growing in mice receiving either PSMA-V_H_-T or MSLN-V_H_-T cells were predominantly MSLN and PSMA expressing cells, respectively (figure 7E). These results indicate that bispecific CARs generated by joining two human Ab V_H_ domains can prevent tumor escape in tumor with heterogeneous antigen expression.

10.1136/jitc-2020-002173.supp4 Supplementary data

10.1136/jitc-2020-002173.supp5 Supplementary data

## Discussion

CARs approved by the Food and Drug Administration and those in clinical studies are mostly based on scFv-binding moieties. Here we demonstrated that monospecific human V_H_ domain-based CAR-T cells achieved comparable antitumor effects both in vitro and in vivo as scFv-based CAR-T cells. Furthermore, V_H_ domains combined in tandem to create bispecific molecules allowed the generation of effective CAR-T cells targeting two antigens.

Redirected T cell based on single-domain Abs have been recently proposed.^17 28 29^ However, most of them are obtained from llamas or camelid-derived libraries. Biological therapeutic molecules with non-human sequence can cause immune responses.^18 28^ Transgenic mouse technology has enabled the generation of biophysically robust fully human V_H_ domains known as Humabody V_H_ or Humabodies^30^ which have the potential for use in CAR constructs while mitigating immunogenicity risk.

Despite the remarkable clinical activity of CAR-T cells in hematological malignancies, objective responses in patients with solid tumors are modest.^10 26 31–33^ Heterogeneity of antigen expression is one of the main reasons causing tumor escape in solid tumors after targeted therapies.^10 19 20^ Furthermore, murine-based scFv may cause immune responses especially in solid tumor patients who are usually less immunosuppressed compared with patients with liquid tumors. Targeting multiple TAAs and using human binding moiety in CAR molecules may improve the outcome of CAR-T cells in solid tumors.^10^ Here, we demonstrated that human V_H_ domains generated from a transgenic mouse might solve both issues of immunogenicity and tumor heterogeneity since bispecific CAR-T cells can be efficiently generated using two human V_H_ domains in tandem.

In addition to the issue of heterogeneity in antigen expression, the complex inhibitory pathways of the tumor microenvironment in solid tumors mean that additional genetic modification of T cells would likely be required to enhance T cell trafficking and functions.^5 31 34–36^ Generation of vector cassettes encoding multiple genes requires a significant optimization of the engineering strategies since the size of the entire cassette is limited. V_H_ domains are a good alternative to scFv since they are approximately half the size.

Here, we have used two target antigens, PSMA and MSLN, that are currently under evaluation to treat mesothelioma, lung cancer, breast cancer, pancreatic cancer and prostate cancer via scFv-based CAR-T cells.^37–39^ Our preclinical experiments validate the potential use of bispecific human V_H_ domains targeting both PSMA and MSLN in these difficult to treat malignancies. It remains to be validated if dual or multiple targeting with V_H_ domain-based CARs can be broadly applicable, and if targeting multiple antigens in solid tumors leads to increased potential for toxicity.

Additionally, we observed that V_H_ domain-based CAR-T cells have comparable cytotoxicity and proliferative capacity as traditional scFv-based CAR-T cells. MSLN-V_H_-T cells showed even more profound antitumor effects as compared with mice treated with MSLN-scFv CAR-T cells. Interestingly, MSLN-V_H_ showed lower affinity than MSLN-scFv (28 nM compared with 79pM) recapitulating what has been observed for other scFvs that very high affinity is not necessarily optimal for CAR-based targeting for some targets.^40–42^ However, we cannot exclude that the observed superior antitumor activity of the MSLN-V_H_-based CAR-T cells can be associated with the recognition of a different epitope rather than to different affinity. In summary, we have demonstrated that V_H_ domain CAR-T cells in monospecific format achieved comparable antitumor response compared with traditional scFv-based CAR-T cells both in vitro and in vivo. Furthermore, bispecific V_H_ domain CAR-T cells delivered potent anti-tumor effects demonstrating the potential to target solid tumors with heterogeneous antigen expression. These proof-of-concept experiments lay the foundation for further development of human V_H_ domain-based CAR-T cells in clinical trials.

## Data Availability

All data relevant to the study are included in the article or uploaded as supplementary information.
