# Supplementary figures and images for: Fully human antibody VH domains to generate mono and bispecific CAR to target solid tumors
Source: J Immunother Cancer. 2021 Apr 1;9(4):e002173. doi: 10.1136/jitc-2020-002173 (PMC8021891; doi:10.1136/jitc-2020-002173)

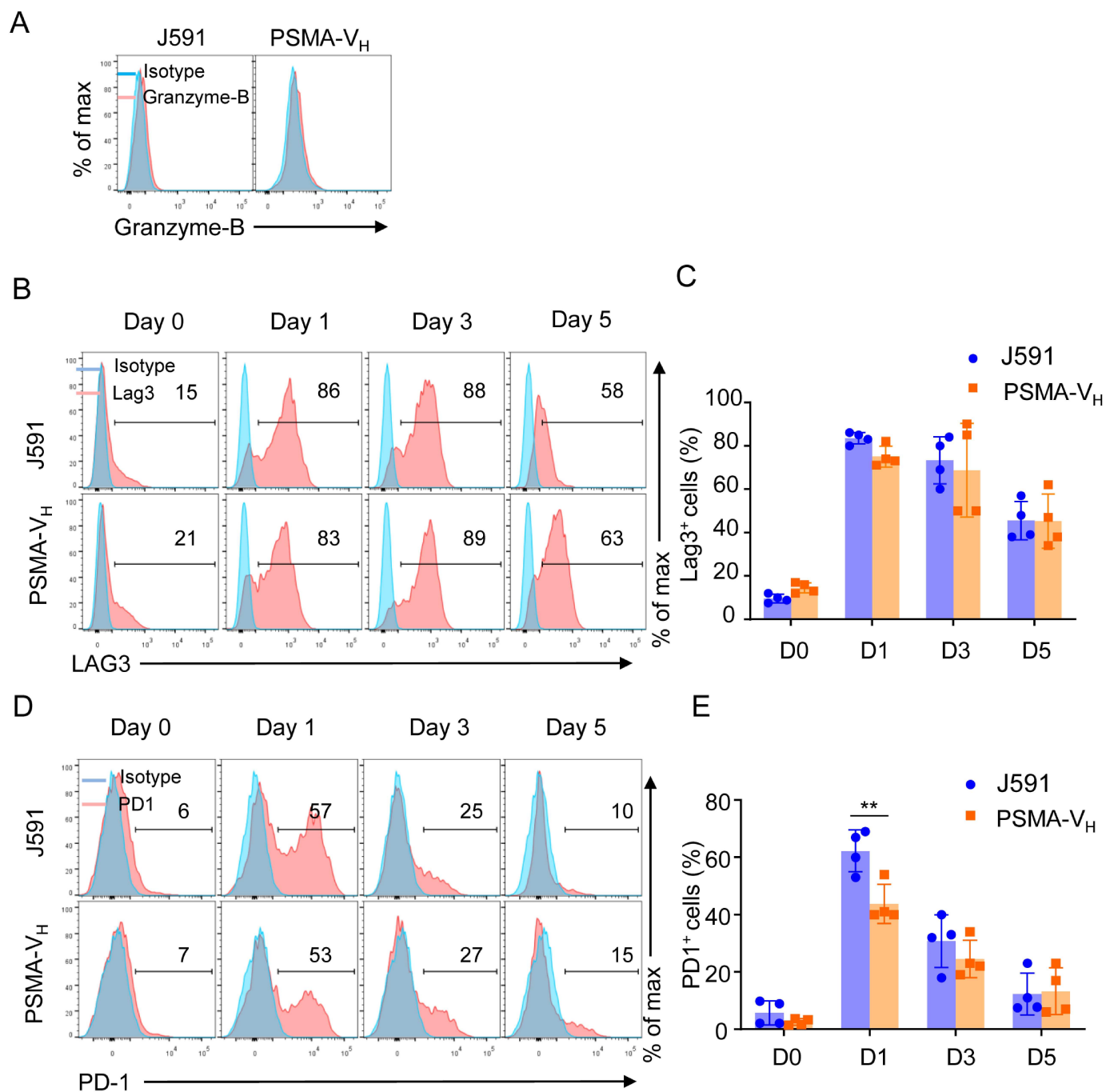

Supplement: Supplementary data [file jitc-2020-002173supp001.pdf]

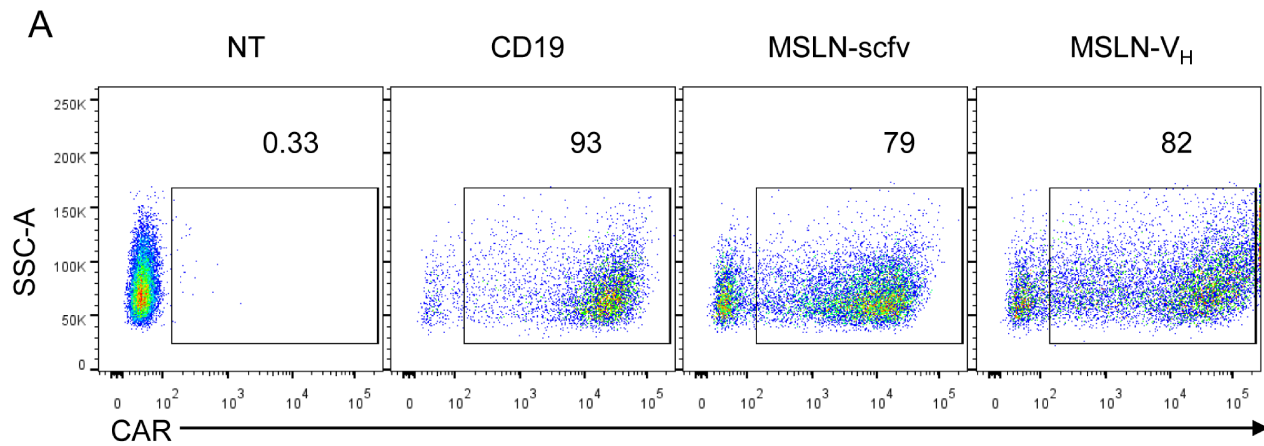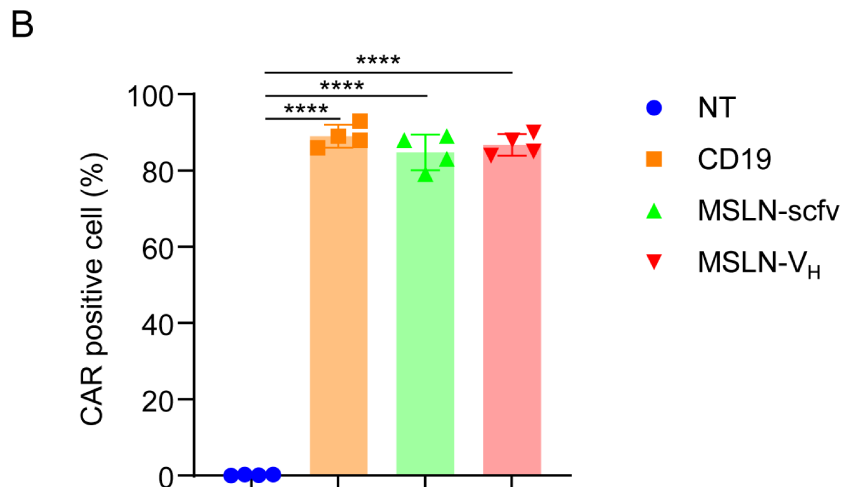

Supplement: Supplementary data [file jitc-2020-002173supp002.pdf]

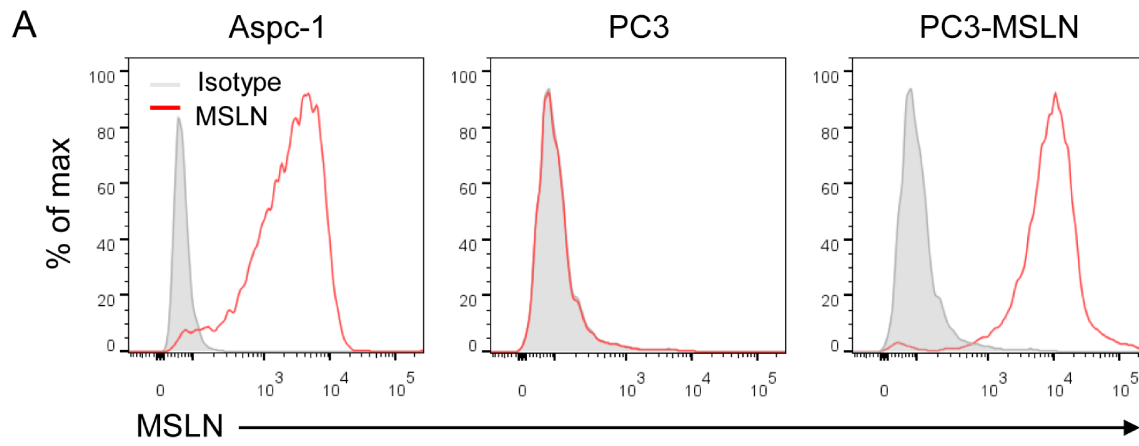

Supplement: Supplementary data [file jitc-2020-002173supp003.pdf]

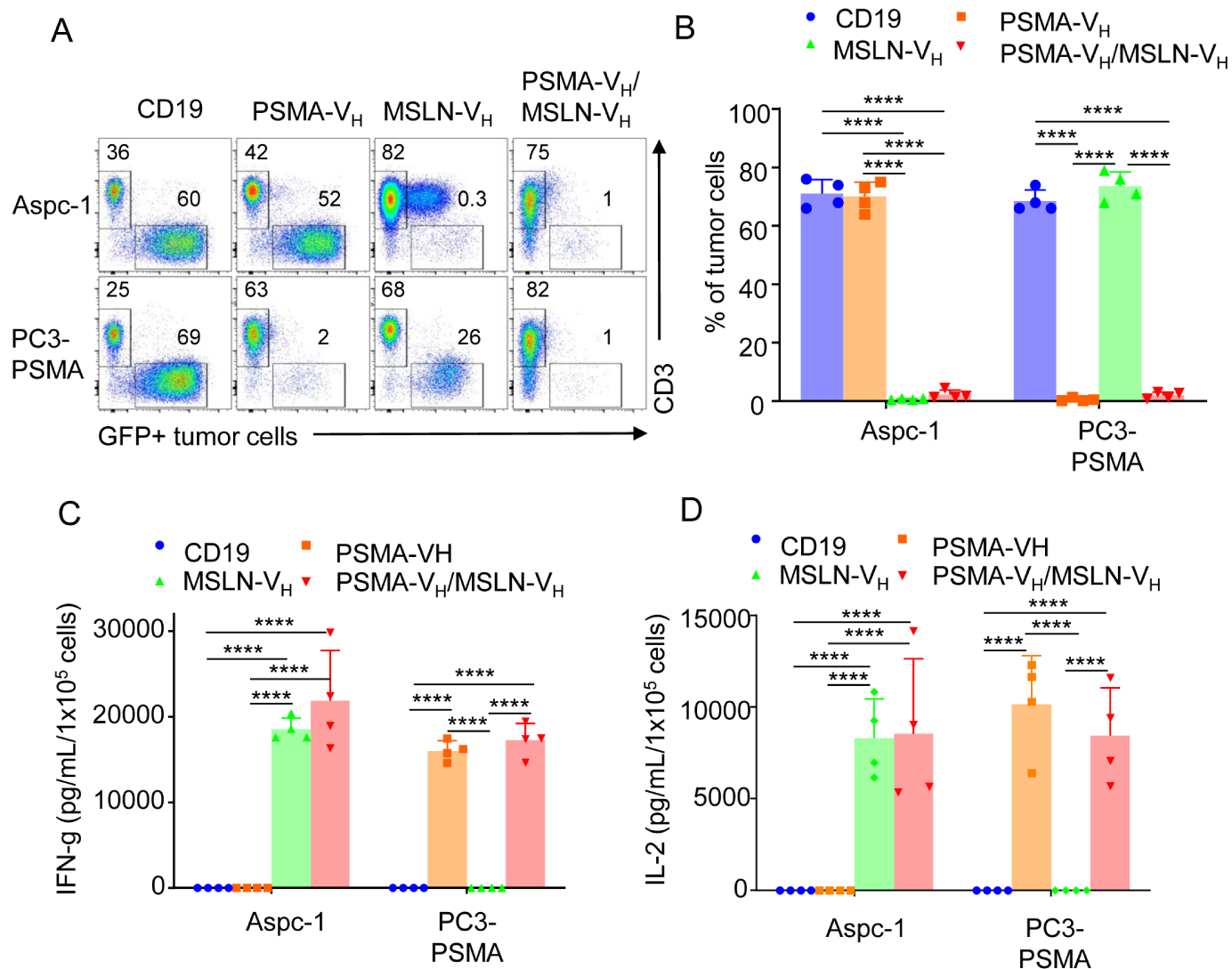

Supplement: Supplementary data [file jitc-2020-002173supp004.pdf]

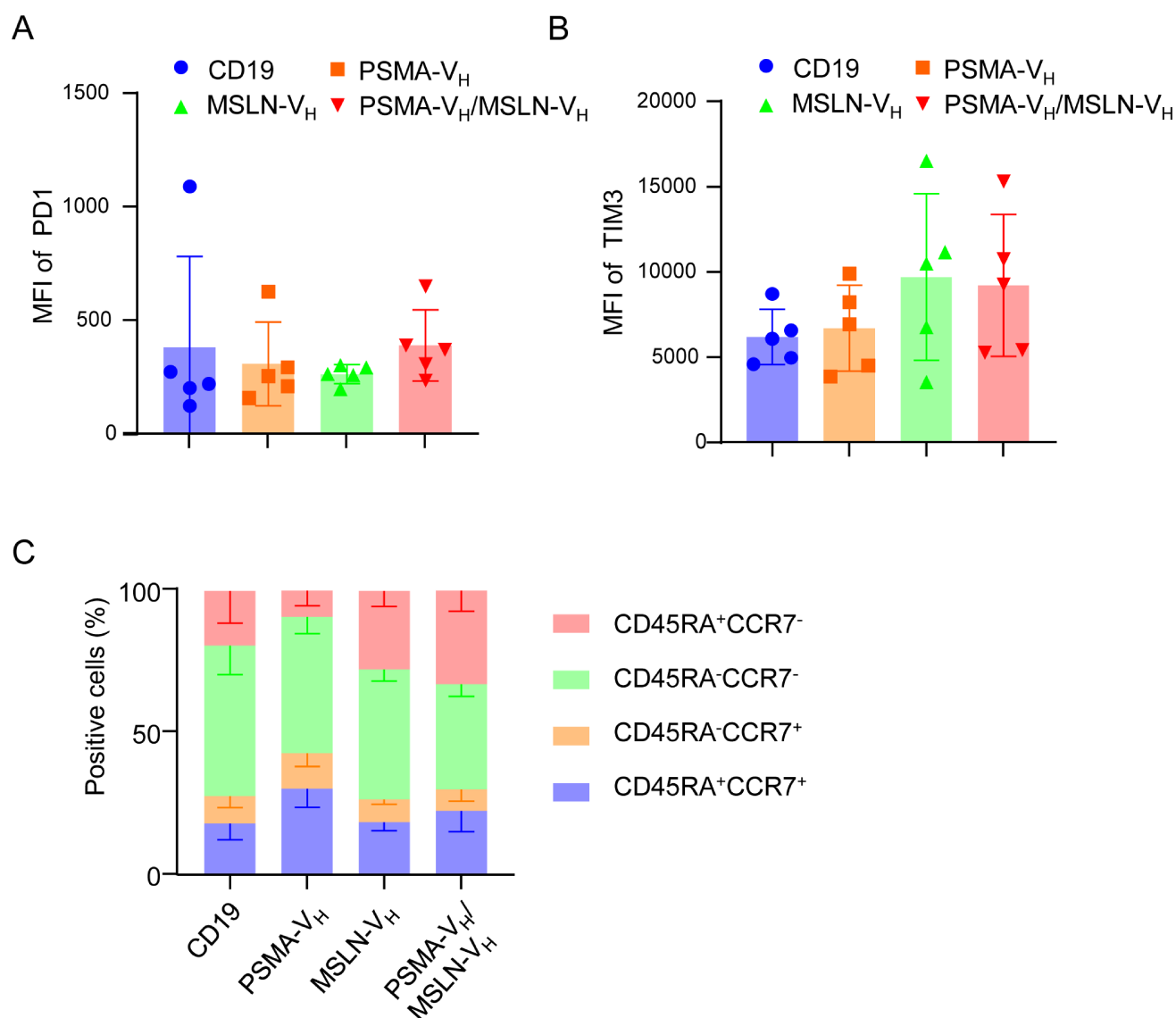

Supplement: Supplementary data [file jitc-2020-002173supp005.pdf]
