# Supplementary material for: Fully human antibody VH domains to generate mono and bispecific CAR to target solid tumors
Source: J Immunother Cancer. 2021 Apr 1;9(4):e002173. doi: 10.1136/jitc-2020-002173 (PMC8021891; doi:10.1136/jitc-2020-002173)
Supplement: Supplementary data [file jitc-2020-002173supp006.pdf]

Figure S1. Heavy-chain-only-based CAR-T cells express LAG3 and PD-1 upon activation as scFv-based CAR-T cells. (A) Representative flow cytometry plots illustrating Granzyme-B expression in T cells expressing either J591 or PSMA-VH without co-culture with tumor cells (rest condition). (B-E) Representative flow plots and summary illustrating the kinetics of LAG3 (B,C) and PD-1 (D,E) expression in T cells expressing either J591 or PSMA-VH cocultured overnight with the tumor cell line expressing PSMA (PC3-PSMA-eGFP) at E:T ratio of 1:2. Data are representative of 4 experiments. **p < 0.01 Two-way ANOVA.

Figure S2. Transduction efficiency of MSLN-scFv and MSLN-VH CARs. (A,B) Representative flow pots (A) and summary (B) illustrating MSLN-scfv and MSLN-VH expression in T cells. The CD19-specific CAR (CD19) and non-transduced T cells (NT) were used as positive and negative controls, respectively. ****p < 0.0001, One-way ANOVA.

Figure S3. Expression of MSLN in Aspc-1, PC3 and engineered PC3 cells. Representative flow cytometry histograms showing the expression of MSLN in Aspc-1, PC3 and PC3 cells engineered with retroviral vector to express MSLN.

Figure S4. Bispecific heavy-chain-only-based CAR-T cells demonstrate dual specificity. (A) Representative flow cytometry plots showing coculture of PC3-PSMA-eGFP (PSMA target) and Aspc-1-eGFP (MSLN target) tumor cells with CD19.CAR, PSMA-VH.CAR, MSLN-VH.CAR and PSMA-VH/MSLN-VH.CAR T cells at E:T ratio of 1:5 for 6 days. At the end of co-culture, cells were collected to enumerate T cells (CD3) and tumor cells (GFP), respectively by flow cytometry. (B)

Summary of coculture experiments illustrated in (A); error bars represent SD, (n = 4). ****p < 0.0001, Two-way ANOVA. (C,D) IFN-γ (C) and IL-2 (D) released in the coculture supernatant of the experiments illustrated in (A) as measured by ELISA; error bars represent SD, (n = 4). ****p < 0.0001, Two-way ANOVA.

Figure S5. Phenotypic characterization of T cells in the peripheral blood of mice in the PC3-PSMA and PC3-MSLN Mixed tumor model. CAR-T cells in the peripheral blood at day 21 post second T-cell infusion were identified by the expression of CD45 and CD3 by flow cytometry. PD1 (A), TIM3 (B), CD45RA and CCR7 expression (C) were examined, error bars represent SD, (n = 5). p ≥0.05 by One-way ANOVA.
